# The impact of community-based, peer-led sexual and reproductive health services on knowledge of HIV status among adolescents and young people aged 15 to 24 in Lusaka, Zambia: The Yathu Yathu cluster-randomised trial

**DOI:** 10.1371/journal.pmed.1004203

**Published:** 2023-04-21

**Authors:** Bernadette Hensen, Sian Floyd, Mwelwa M. Phiri, Ab Schaap, Lucheka Sigande, Melvin Simuyaba, Lawrence Mwenge, Rosemary Zulu-Phiri, Louis Mwape, Sarah Fidler, Richard Hayes, Musonda Simwinga, Helen Ayles

**Affiliations:** 1 Department of Public Health, the Institute of Tropical Medicine, Antwerp, Belgium; 2 Department of Clinical Research, the London School of Hygiene and Tropical Medicine, London, United Kingdom; 3 Department of Infectious Disease Epidemiology, the London School of Hygiene and Tropical Medicine, London, United Kingdom; 4 Zambart, Lusaka, Zambia; 5 Department of Infectious Disease, Imperial College London, United Kingdom; University of Washington, UNITED STATES

## Abstract

**Background:**

The growing population of adolescents and young people (AYP) aged 15 to 24 in sub-Saharan Africa face a high burden of HIV in many settings. Unintended pregnancies among adolescent girls in the region remain high. Nonetheless, the sexual and reproductive health (SRH) service needs of AYP have remained underserved. We conducted a cluster-randomised trial (CRT) to estimate the impact of community-based, peer-led SRH service provision on knowledge of HIV status and other SRH outcomes, including met need for contraceptives.

**Methods and findings:**

Yathu Yathu was a cluster-randomised trial (CRT) conducted from 2019 to 2021 in 2 urban communities in Lusaka, Zambia. The communities were divided into 20 zones (approximately 2,350 AYP/zone) that were randomly allocated to the Yathu Yathu intervention or control arm. In each intervention zone, a community-based hub, staffed by peer support workers, was established to provide SRH services. In 2019, a census was conducted in all zones; all consenting AYP aged 15 to 24 were given a Yathu Yathu card, which allowed them to accrue points for accessing SRH services at the hub and health facility (intervention arm) or the health facility only (control arm). Points could be exchanged for rewards, thus acting as an incentive to use SRH services in both arms. We conducted a cross-sectional survey in 2021 to estimate the impact of Yathu Yathu on the primary outcome: knowledge of HIV status (self-reporting living with HIV or HIV testing in the last 12 months) and secondary outcomes, including use of pre-exposure prophylaxis (PrEP) in the last 12 months, current use of antiretroviral therapy (ART), and met need for contraceptive services. The sampling was stratified on sex and age group, and we analysed data at cluster-level using a two-stage process recommended for CRTs with <15 clusters/arm. A total of 1,989 AYP consented to participate in the survey (50% male); consent was similar across arms (63% consent/arm). Across zones, knowledge of HIV status ranged from 63.6% to 81.2% in intervention zones and 35.4% to 63.0% in control zones. Adjusting for age, sex, and community, knowledge of HIV status was higher in the intervention arm compared to control (73.3% versus 48.4%, respectively, adjusted prevalence ratio (PR) 1.53 95% CI 1.36, 1.72; *p* < 0.001). By age and sex, results were similar. There was no evidence for impact on any secondary outcomes, including current use of ART and met need for contraceptives. There were no adverse events reported in either arm. A key limitation of our trial is that approximately 35% of the AYP randomly selected for participation in the endline survey could not be reached.

**Conclusions:**

Delivering community-based, peer-led SRH services increased knowledge of HIV status among AYP, both males and females, compared with the control arm. Scaling up the highly effective Yathu Yathu strategy has the potential to make a substantial contribution to increasing access to HIV prevention and care services for young people. However, additional implementation research is needed to understand how to improve uptake of broader SRH services, beyond uptake of HIV testing.

**Trial registration:**

ISRCTN75609016, clinicaltrials.gov number NCT04060420

## Abstract 2

Bernadette Hensen and colleagues investigate whether community-based delivery of sexual and reproductive health services by peers increased knowledge of HIV status among adolescents and young people in Zambia.

## Author summary

**Why was this study done?:**

- Adolescents and young people (AYP) have limited access to sexual and reproductive health (SRH) services; rigorous evidence of strategies, co-developed with young people, that improve access to these services is needed.
- This trial was done to investigate whether community-based delivery of SRH services by peers would increase knowledge of HIV status among AYP aged 15 to 24 in Lusaka, Zambia.

**What did the researchers do and find?:**

- Through spaces in the community (called hubs), peer support workers, a nurse and supervisor, delivered a range of SRH services, including HIV testing, comprehensive sexuality education, and contraceptives, daily to AYP aged 15 to 24.
- Service delivery through hubs was complemented by incentives, namely the collection of points for services accessed and the ability to use these points to “buy” rewards, such as soap, toothbrush, and toothpaste.
- After approximately 24 months, we compared knowledge of HIV status (defined as either self-reporting knowing ones HIV–positive status or having HIV tested in the previous 12 months) between random selections of AYP aged 15 to 24 (*N* = 1,989) living in areas that received the Yathu Yathu intervention and in areas that did not receive the intervention.
- We found that knowledge of status was considerably higher in the intervention areas (73.3%) as compared to the non-intervention areas (48.4%), but that there was no difference in uptake of other SRH services, such as linkage to PrEP for the prevention of HIV.

**What do these findings mean?:**

- These findings show that community-based hubs that deliver a range of SRH services and are complemented by incentives can increase knowledge of HIV status.
- Additional research is needed to improve delivery of other services, including linkage to prevention and care services, and access to contraceptive services.

## Introduction

Across sub-Saharan Africa, the population of adolescents and young people (AYP) aged 15 to 24 years is growing rapidly. By 2030, it is estimated that youth aged 15 to 24 will account for 26% of the total African population [1]. This growing population faces a high burden of HIV, with adolescent girls and young women (AGYW) in the region accounting for 24% of new HIV infections in 2019 despite accounting for only 10% of the population [2]. Relative to older adults, youth have limited access to HIV-related services and poorer outcomes across the HIV care continuum, and AIDS remains a leading cause of mortality among youth [3,4]. Among adolescent girls aged 15 to 19, pregnancy and childbirth complications are a leading cause of mortality globally; in 2017, countries in sub-Saharan Africa and Southern Asia accounted for 86% of maternal deaths globally [5]. In parallel with the growing population of youth, there will likely be increased numbers of unintended or mistimed pregnancies among adolescent girls aged 15 to 19 [6]. Considering the synergies between HIV and sexual and reproductive health (SRH) more broadly, integrating the delivery of HIV services with broader SRH services may be helpful in meeting the SRH needs of AYP and optimising uptake of SRH services [7].

In Zambia, there were an estimated 4 new HIV infections per 1,000 uninfected people aged 15 to 24 in 2021 [8], the seventh highest incidence among youth globally; Zambia also had the seventh highest HIV prevalence [8]. According to the 2018 Demographic and Health Survey, HIV prevalence among AGYW was 5.6% compared to 1.8% among adolescent boys and young men [9]. An estimated 59% of female and 43% of male youth tested for HIV in the previous 12 months, considerably lower than the corresponding figures (72% and 65%, respectively) among adults aged 25 to 29 years [9]. In addition to a high burden of HIV, Zambia had the 11th highest adolescent (aged 15 to 19) fertility rate globally in 2020 [10]; in 2018, 29% of adolescent girls reported at least 1 lifetime pregnancy with little change from 34% in 1992 [9]. In Lusaka, however, adolescent pregnancies reduced substantially—from 31% in 1992 to 15% in 2018 [9,11]. During 2020 to 2021, measures to control the spread of Coronavirus Disease 2019 (COVID-19), including physical distancing, orders to stay home where possible, and restrictions on public gatherings, were implemented nationally. These measures, alongside indirect effects of fear of infection and mandatory COVID-19 testing at health facilities, likely further constrained access to available SRH services among youth.

Improving the SRH of youth is reflected in Sustainable Development Goal 3, target 3.7 [12], which aims to achieve universal access to SRH services by 2030. With youth underserved by available SRH services [13], failure to improve coverage of SRH services among AYP will likely mean they will continue to experience poor SRH outcomes, with consequences for the physical, social, and economic development of a growing population. To date, interventions to increase coverage of SRH services among youth have largely focussed on improving youth-friendly services at health facilities or on school-based delivery of information and education [14,15]. There is evidence that interventions that improve service delivery at health facilities in combination with demand creation and/or in-school education have an impact on SRH knowledge and attitudes [16,17], self-reported pregnancies [16], and condom use [18]. Additionally, a systematic review of 31 school-based interventions found an impact on self-reported condom use [15]. In Kenya, one of the countries where the DREAMS partnership was implemented, the package of interventions included providing HIV testing services to AGYW at “safe spaces” in the community; AGYW who were invited to participate in DREAMS had substantially higher knowledge of their HIV status compared with those not invited to participate [19]. However, there are few randomised trials of interventions to improve coverage of SRH services among youth, with fewer still evaluating the impact of out-of-school, community-based strategies [14,15].

Calls for meaningful engagement of youth in research, including research to develop strategies to improve health and well-being, have led to an increase in their involvement; however, youth involvement in the design and evaluation of strategies to improve their SRH remains limited [20–22]. Improving the SRH outcomes of youth requires strategies that meet the needs and preferences of youth, which in turn requires engaging them in the design and delivery of services [20]. As such, there remains a need for evidence of whether strategies designed with and also delivered by youth could complement existing SRH services to address the limitations of existing systems. In response, during 2018 to 2019, we co-designed, with youth, a strategy to deliver universal, comprehensive, community-based, and peer-led SRH services to AYP aged 15 to 24 in Lusaka, Zambia [23]. The strategy, called Yathu Yathu, built on consultations with AYP to understand their needs, participatory research, and lessons learned through the HPTN-071 (PopART) trial and the nested P-ART-Y study [24,25]. These studies found that home-based delivery of a universal HIV testing and treatment intervention reached youth, with the first 90 of the UNAIDS 90-90-90 target (90% of individuals living with HIV knowing their HIV–positive status) almost reached among adolescents [25], but that there were gaps in reaching younger men and challenges in sustaining high coverage of services among youth [23,26–28]. Reaching the UNAIDS 95-95-95 targets (95% of individuals living with HIV knowing their HIV–positive status, 95% of people who know their HIV–positive status on treatment, and 95% of HIV–positive individuals on treatment achieving viral suppression) and achieving the goal of HIV elimination requires a concerted effort to reach AYP with HIV testing and linkage to treatment and prevention services.

Yathu Yathu combined the delivery of comprehensive SRH through community-based spaces (hubs) with a prevention points “loyalty” card system to incentivise service use. Being a community-level intervention, the impact of Yathu Yathu was evaluated using a cluster-randomised trial (CRT) design. We anticipated that comprehensive SRH services, including HIV services, developed for youth and delivered by youth, combined with incentives would facilitate progress towards the revised UNAIDS 95-95-95 targets among AYP and support AGYW with access to contraceptive services. In this paper, we present the results of the CRT to evaluate the impact of Yathu Yathu on the primary outcome of knowledge of HIV status as well as its impact on secondary HIV- and SRH-related health outcomes, including uptake of medical male circumcision and met need for family planning services.

## Methods

This study is reported according to the CONSORT extension for Cluster-Randomised Trials (S1 CONSORT Checklist) [29].

### Study location

Details of the Yathu Yathu trial are described elsewhere [30] (see https://researchonline.lshtm.ac.uk/id/eprint/4663006/). Briefly, Yathu Yathu was a parallel-arm CRT conducted in 2 peri-urban communities in Lusaka, Zambia. Both communities were included in the HPTN 071 (PopART) trial and had received the PopART intervention. Across the 21 communities, the HIV incidence from 2015 to 2018 was approximately 2 per 100 among women aged 18 to 24 and approximately 0.7 per 100 among men aged 18 to 24.

Within these 2 communities, 20 geographical zones (clusters) of approximately equal population size were demarcated. Where possible, demarcation followed natural boundaries, including major roads. Each of the 20 zones had an estimated population of approximately 2,350 adolescents and young people aged 15 to 24.

### Study design and participants

Between August and September 2019, prior to the implementation of the Yathu Yathu strategy, a census of all households was conducted in all 20 clusters. During the census, all enumerated AYP were offered a prevention points card (PPC). In zones randomly allocated to the Yathu Yathu trial arm, AYP were informed that they could use their PPC to collect points for accessing SRH services at the hub in their zone or the local health facility and that these points could be used to redeem rewards. In control zones, AYP were informed that they could use their PPC to collect points for accessing SRH services at the local health facility and that these points could be used to redeem rewards. In addition to incentivising service access, the PPC data were used to monitor service use and estimate service coverage [30,31].

After completion of the census, AYP who were not in possession of a PPC could still access services at the hubs but were not added to the enumeration list. Inclusion on the enumeration list was restricted due to the inability to accurately verify whether the AYP resided in a control or intervention zone. As such, the AYP enumerated at the start of the study period formed a “closed cohort” of AYP, among whom study outcomes were measured using PPC data and through a cross-sectional “endline” survey.

### Randomisation and masking

We used restricted randomisation, stratified by community, to balance the 2 trial arms on: participation in the PopART intervention during the last year (2017) of delivery, knowledge of HIV status among AYP who participated in the PopART intervention during its last year, uptake of HIV testing among AYP who participated in the PopART intervention during its last year, average population of AYP per zone, and distance from the centre of the zone to the local health facility [30] and randomly allocated the 20 zones, in a 1:1 allocation ratio, to the Yathu Yathu or control arm. Randomisation was finalised in a public ceremony with members of the 2 study communities. Details of the randomisation are described elsewhere [30]. Due to the nature of the Yathu Yathu strategy, it was not possible to mask study communities or teams as to whether a zone had been allocated to the Yathu Yathu or control arm. The statistical analysis plan was finalised prior to analysis of data from the cross-sectional survey that was conducted to measure primary and secondary outcomes.

### Procedures—The Yathu Yathu strategy

**Fig 1:**
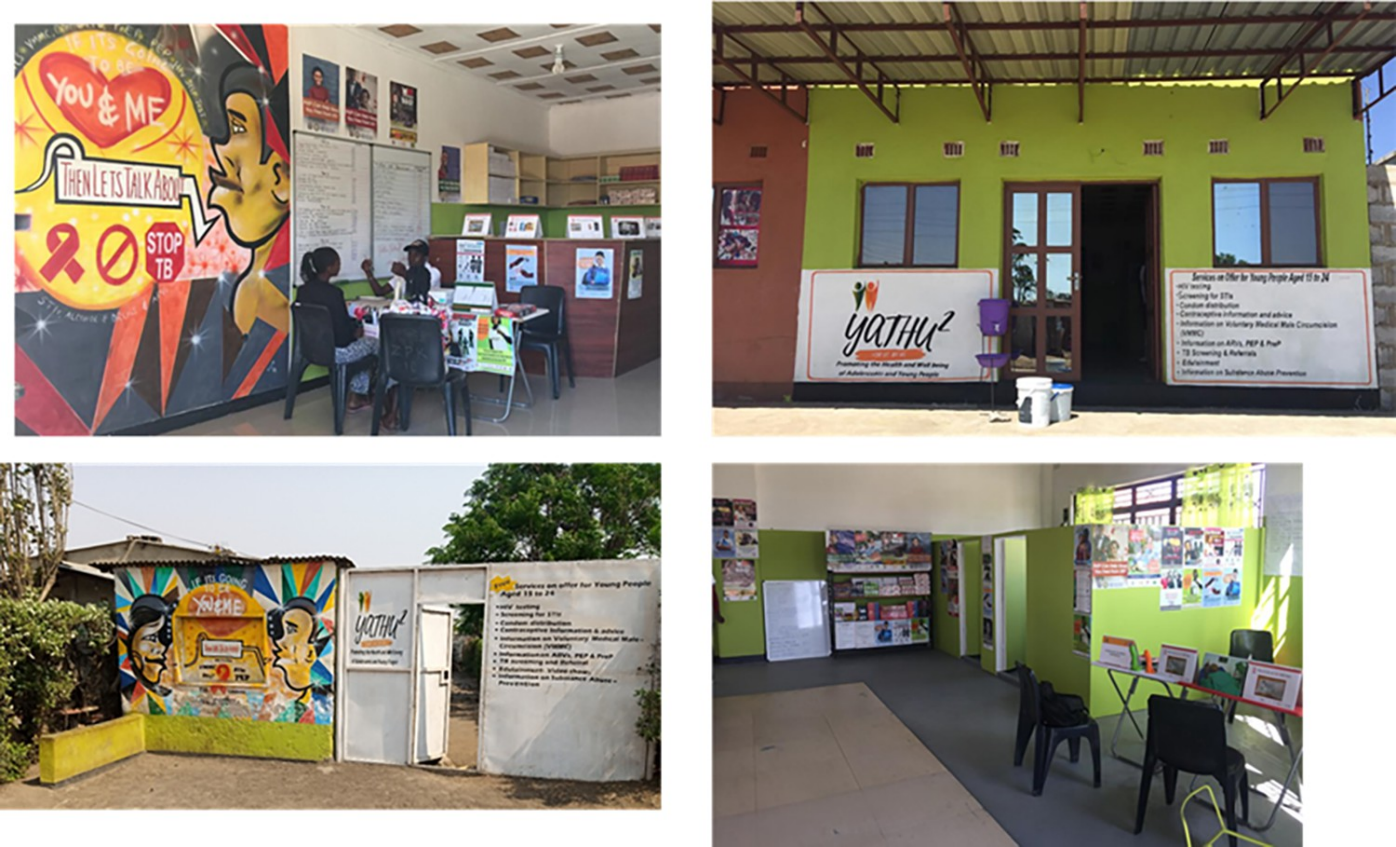
Pictures of the Yathu Yathu hubs.

Between September 2019 and September 2021, the Yathu Yathu strategy was implemented in the 10 randomly selected intervention clusters. Yathu Yathu consisted of 2 key components: (1) community-based delivery of comprehensive SRH services through hubs (Fig 1); and (2) the PPC to incentivise service use. Services available at the hubs included: HIV testing, including oral HIV self-testing, counselling and referrals appropriate to the result of the HIV test, contraceptives (including the pill and injectables, with referrals to the health facility for implants and IUDs), condom promotion and distribution, comprehensive sexuality education and edutainment sessions (MTV Shuga and Love Games) [30,32]. Yathu Yathu did not offer pre-exposure prophylaxis (PrEP), antiretroviral therapy (ART), or voluntary medical male circumcision (VMMC) services on site at the hubs, but referred AYP to the local health facility for these services.

**Fig 2:**
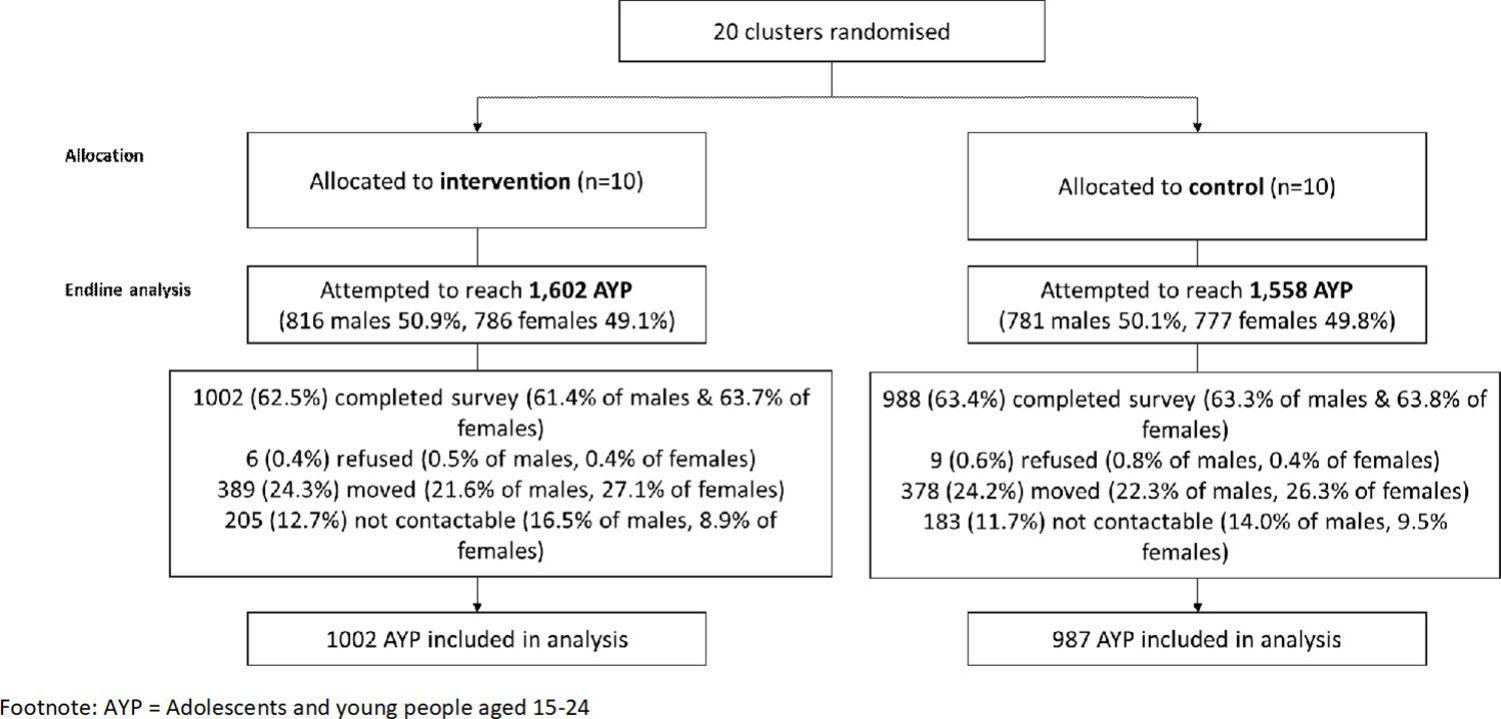
Flowchart of participation in the endline survey.

For each of the services accessed, AYP could collect points on their PPC. These points could then be used to “purchase” a reward. Rewards were primarily health-related items, such as toothpaste, toothbrush, and soap, but also nail polish, vouchers for barber/hairdresser, and reusable menstrual pads [30]. The PPC had the added advantage of enabling uptake of services to be monitored in real time, which facilitated adaptation of the Yathu Yathu strategy following a pilot phase and subsequent ongoing adaptation and evaluation of its impact on uptake of SRH services [32]. Alongside service delivery and incentives, community engagement formed an integral component of the intervention. In particular, community dialogue meetings with parents and guardians (*n* = 14) were important to inform them of activities and address concerns regarding delivery of SRH services to adolescents.

We developed a logic model to inform how we anticipated Yathu Yathu to impact the primary and secondary outcomes [30]. We anticipated that youth-friendly hubs, managed by peer support workers, would be acceptable spaces for AYP to access SRH services and information, that they would have positive experiences at the hubs, leading to more regular attendance, and that the incentives would lead to AYP placing greater value on SRH services. Results from an embedded process evaluation will be published separately.

During the implementation period, all 10 hubs were closed between April and July 2020 in response to COVID-19 [32]. Upon reopening, service delivery through the hubs was modified for safety reasons. These modifications were decided and implemented by the study team. Face masks and handwashing were required before entering the hubs, in-person comprehensive sexuality education (CSE) was cancelled with sessions delivered via social media platforms, and only one-to-one services offered on site, for example, HIV testing and contraceptives [32]. After 1 month, and following a consultation with AYP and the community, CSE was reinstated, but with restrictions on the number of AYP who could participate per session. Details of adaptations in response to COVID-19 are reported elsewhere [32].

### Procedures—the endline cross-sectional survey

The impact of Yathu Yathu was measured in a cross-sectional survey conducted between April and November 2021. Simple random sampling, stratified by sex and age group (15 to 19 or 20 to 24 years) at the time of the population census, was used to select approximately 100 AYP per cluster (approximately 2,000 AYP across all 20 clusters) with equal target sample size for each of the 4 age/sex groups. Details of the survey are described elsewhere [30]; briefly, AYP were selected from the list of AYP aged 15 to 24 years who were enumerated and given a PPC at the start of the study. During the survey, research staff visited the households of individuals randomly selected to participate in the survey, with up to 3 household visits conducted. If, after 3 household visits, the selected AYP was not met they were recorded as not contacted.

AYP who were contacted and consented to participate completed a questionnaire with questions on: sociodemographic and socioeconomic variables, knowledge of HIV status and uptake of HIV testing, uptake of HIV treatment and prevention services, alcohol use, history of contraceptive use and pregnancy, condom use, and the Hope scale (to measure AYP’s expectations for the future) [33]. The questionnaire was completed on a personal digital assistant and administered by a research assistant. For questions on sexual behaviours, AYP could choose to self-complete the questions. As part of the survey, finger-prick HIV testing was offered as an opportunity for AYP to learn their HIV status. HIV testing was conducted according to Zambian national guidelines and performed by individuals trained in offering and conducting HIV testing. Confirmatory testing of a positive test result was conducted using a second HIV test. As HIV testing was primarily being offered as a service, an individual who did not consent to HIV testing was still eligible to participate in the survey through questionnaire completion.

### Primary and secondary outcomes

The primary outcome of the CRT was self-reported knowledge of HIV status (self-report HIV–positive status or HIV testing in the past 12 months). The denominator was the number of AYP who participated in the survey and reported on their history of HIV testing. The numerator was the number of AYP who self-reported that they were living with HIV or that they had tested HIV–negative in the previous 12 months, and the outcome was the proportion of AYP who knew their HIV status.

Secondary outcomes included the following, all measured as proportions: (1) the proportion of adolescent boys and young men (ABYM) who accessed VMMC services since Yathu Yathu was first implemented, among those who were not circumcised prior to implementation of Yathu Yathu; (2) the proportion of individuals who reported using PrEP in the previous 12 months, among those who self-reported they were HIV–negative; (3) the proportion of individuals who reported they were currently on ART, among those who self-reported they were living with HIV; (4) the proportion of AGYW who reported they were currently using a contraceptive method, among those who reported they had a sexual partner in the previous 12 months and that they did not want (more) children or that they wanted to delay having children for 1 to 2 years or that they were unsure about when they wanted to have a child; and (5) the proportion of all AGYW who reported a birth in the last 6 and last 12 months, overall and with restriction to those who reported they had ever had sex.

An additional secondary outcome was the coverage of key SRH services, measured using the PPC data. This key secondary outcome, alongside data on reach and services accessed, will be reported in a manuscript lead by MP and AS [31].

### Data analysis

As described elsewhere [30], the study was powered to detect an approximately 15% difference in the proportion of AYP with the primary outcome, comparing the Yathu Yathu and control arms. With 10 clusters per arm, we used a “two-stage” cluster-level analysis method that is appropriate for the analysis of CRTs with <15 clusters per arm [34–36].

Our analysis was designed prospectively and proceeded in 3 steps [30]. First, we described participation in the study, at cluster and individual levels, and the characteristics of AYP who participated in the survey. Overall and by trial arm, we described the number and proportion of individuals contacted; among individuals not contacted, we described the reason they were not contacted; and among those who were contacted, we described the number and percentage of individuals who consented to participate in the survey. We assessed whether trial arms were balanced in terms of education and marital status, as these characteristics were found to be associated with uptake of HIV testing services in an analysis of Yathu Yathu pilot data [37]. Where there was imbalance, these variables were adjusted for in sensitivity analyses, as described below. Subsequently, we described among survey participants, by trial arm, characteristics that are not expected to have been changed due to the Yathu Yathu intervention, including age, sex, education, marital status, and household economic status.

Secondly, for each of the 20 clusters, the proportion of survey respondents with knowledge of their HIV status was calculated. We calculated the average value of the cluster-specific proportions of individuals who reported knowledge of their HIV status for each trial arm, using both the arithmetic and geometric means. We also estimated the coefficient of variation (k). Next, we calculated the prevalence ratio (PR), comparing the Yathu Yathu arm with the control arm, based on the ratio of the geometric means for each trial arm. To formally compare the trial arms in an “unadjusted” analysis, we then fitted a linear regression model of log (cluster-level proportion) on trial arm and community, in order to obtain a log(PR) comparing the Yathu Yathu arm with the control arm and a corresponding 95% confidence interval.

In the third step, we followed the two-stage procedure for analysis of CRTs with <15 clusters per arm, in order to adjust for any imbalance between trial arms in survey participants’ sex and age group (and in sensitivity analysis, for additional individual-level characteristics) [34–36]. In Stage 1, a logistic regression model, which included community, and the 4 combinations of age group (15 to 19 or 20 to 24 years at enumeration) and sex, was fitted to the individual-level data. Using this model, we predicted each individual’s probability of knowing their HIV status, under the null hypothesis of no effect of the Yathu Yathu intervention. We aggregated these individual-level probabilities by cluster, to estimate cluster-specific expected numbers of individuals with knowledge of their HIV status under the null hypothesis. In Stage 2, for each cluster, we calculated the ratio of the observed (O) to the expected (E) number of individuals with knowledge of their HIV status (O/E) and calculated the log(ratio-residual) as log(O/E). We then fitted a linear regression model of log(O/E) on trial arm and community to obtain a PR comparing the Yathu Yathu and control trial arms adjusted for community, sex, and age group.

We used the same methods to compare the secondary outcomes between the 2 trial arms. We also conducted prespecified subgroup analysis for each of the 4 sex and age group combinations (males and females aged 15 to 19 and 20 to 24 years). The two-stage analysis described above was repeated, but without adjustment for sex or age group at Stage 1. For the primary outcome, to explore whether there was statistical evidence for interaction by age and sex, we conducted an interaction test.

### Sensitivity analyses

We conducted 2 sensitivity analyses for the primary outcome of knowledge of HIV status. For the first, we followed the same analysis strategy as described, but we adjusted for educational attainment and marital status as these were found to be associated with a history of HIV testing during the pilot phase of the study [37]. To account for any potential contamination in the control arm, the second sensitivity analysis followed the same steps described above. However, in control clusters, individuals who were categorised as having knowledge of their HIV status and who reported visiting a Yathu Yathu hub were classified as not knowing their HIV status (except for individuals self-reporting knowing their HIV–positive status prior to the implementation of Yathu Yathu), under the assumption that they would not have accessed HIV testing services in the absence of Yathu Yathu.

Finally, we conducted a post hoc analysis, comparing secondary outcomes between AYP in the control arm with the subset of AYP in the intervention arm who reported ever attending a Yathu Yathu hub and/or for whom there was evidence from the PPC data that they had accessed Yathu Yathu services.

### Ethics statement

The University of Zambia Biomedical Research Ethics Committee (UNZA BREC; ref number 007-04-19) and the London School of Hygiene and Tropical Medicine (ref number 17104) approved the Yathu Yathu CRT. All AYP were asked for written informed consent to participate in the endline survey. A waiver of parental consent for AYP aged under 18 years was granted from UNZA BREC and LSHTM, as parents/guardians provided consent during enumeration and PPC distribution and, during the P-ART-Y study conducted in the same communities, CAB and aCAB members asked for waiver of parental consent. The study was approved by the National Health Research Authority, Zambia.

The trial registration numbers are: NCT04060420 https://clinicaltrials.gov/ct2/show/NCT04060420; and ISRCTN75609016; https://doi.org/10.1186/ISRCTN75609016.

## Results

Overall, 40,864 AYP were enumerated in the 20 study clusters (20,772 in intervention and 20,092 in control). Among these AYP, 29,370 (71.9%) consented to participate in Yathu Yathu by accepting a PPC (14,872, 71.6%, in intervention and 14,498, 72.2%, in control). In all 10 clusters randomised to the Yathu Yathu intervention, a hub was established and SRH services were delivered as planned.

For the endline cross-sectional survey, attempts were made to contact 1,602 AYP in the intervention and 1,558 in control zones. Overall, 1,990 AYP consented to participated in the survey, with consent similar across trial arms (63% consenting to participate among all AYP for whom a contact attempt was made). Among the AYP randomly selected for participation, one-quarter (*n* = 767) had moved out of the community or zone in which they were enumerated, and additionally approximately 12% (*n* = 388) were not contactable. However, these proportions were balanced across trial arms (Fig 2). One individual who participated in the survey, but had missing data on age and sex, was excluded from the analysis.

Among AYP who consented to participate in the survey (*N* = 1,989), the trial arms were balanced by age group and sex, and current marital and employment status (Table 1). Half (*n* = 996) the survey participants were male and half (*n* = 1,000) aged 15 to 19 at the time of PPC distribution, following the sampling design that was stratified on sex and age group. The majority of participants (80%; *n* = 1,597) were single and had never been married and 30% (*n* = 604) were currently employed. There was little imbalance between trial arms in education, though a higher proportion of AYP in the control arm reported that they had completed secondary education compared with the intervention arm (34.5%, *n* = 341 versus 28.2%, *n* = 282, respectively).

**Table 1:** Key demographic characteristics of the adolescents and young people participating in the endline survey, by arm, 2021.

	Yathu Yathu arm (*N* = 1,002)		Control arm (*N* = 987)	
	Individual characteristic		Individual characteristic	
	Number	%	Number	%
**Sex**				
Male	502	50.1	491	49.7
Female	500	49.9	496	50.3
**Age**				
15–19	502	50.1	498	50.5
20–24	500	49.9	489	49.5
**Highest level of education**				
None/incomplete primary	44	4.4	47	4.8
Complete primary	90	9.0	76	7.7
Incomplete secondary	526	52.5	489	49.5
Complete secondary	282	28.1	341	34.5
Higher education	60	6.0	34	3.4
**Currently employed**				
No	700	69.9	685	69.4
Yes	302	30.1	302	30.6
**Household SEP** *				
1 –Low	199	20.1	198	20.1
2	228	22.8	223	22.6
3 –Medium	174	17.4	186	18.9
4	183	18.3	198	20.1
5—High	215	21.5	181	18.4
**Marital status**				
Single/never married	802	80.0	795	80.5
Married/cohabiting	192	19.2	185	18.7
Divorced/separated/widowed	8	0.8	7	0.7

*(*n* = 85 missing data); SEP–socioeconomic position.

In the intervention zones, 83.3% (*n* = 835; zone range 59.6% to 94.0%) of AYP reported ever attending a Yathu Yathu hub compared to 6.0% (*n* = 57; zone range 0% to 23.0%) of AYP in control zones. Among AYP in intervention zones who reported ever attending a hub, 25.4% (*n* = 212) attended once, 15.4% (*n* = 129) twice, 7.5% (*n* = 63) 3 times, and almost half (46.6%; *n* = 389) attended more than 3 times.

### Impact on knowledge of HIV status

**Fig 3:**
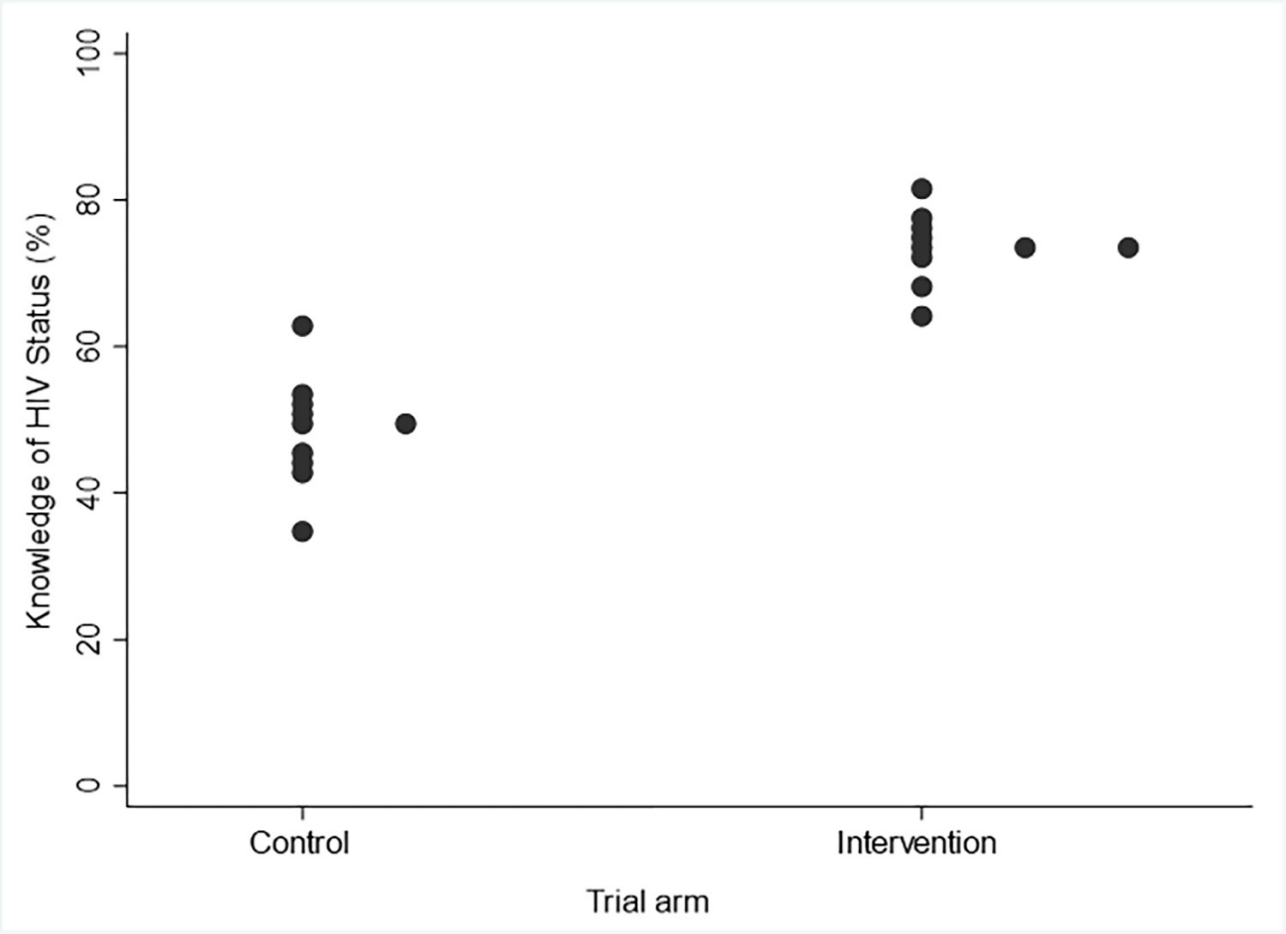
Dotplot showing knowledge of HIV status in each cluster in the intervention arm and control arm. (Note: Dots on the right are shown here to avoid overlap, as they are the same percentage as other dots).

After the implementation period, knowledge of HIV status ranged from 63.6% to 81.2% across intervention and 35.4% to 63.0% across control zones (Fig 3), with an estimated coefficient of variation k of 0.02 in intervention arm communities and 0.11 in control arm communities. Overall, 73.3% (*n* = 735/1,002) of AYP in the intervention arm knew their HIV status compared to 48.4% (*n* = 479/987) in the control arm (adjusted prevalence ratio (adjPR) = 1.53 95% CI 1.36, 1.72; *p* < 0.001; Table 2). The prevalence ratio and its 95% confidence interval were the same without adjustment for age and sex. Results were similar by age and sex (Table 2). However, there was evidence that the intervention had a greater effect among adolescents aged 15 to 19 than among those aged 20 to 24 (*p* = 0.006, test for interaction); in particular, among adolescent boys aged 15 to 19 (*p* = 0.001, test for interaction, comparing adolescent boys versus other individuals for the effect of the intervention). In the intervention arm, 62.2% (*n* = 155/250) of adolescent boys reported knowledge of their HIV status compared to 27.9% (*n* = 70/249) in the control arm (PR = 2.37 95% CI 1.77, 3.17; *p* < 0.001).

**Table 2:** Knowledge of HIV status among adolescents and young people, by arm, 2021.

	Yathu Yathu arm	Control arm	Adjusted PR^2^	95% CI	*p*-value
**Overall**	73.3%^3^ (*n* = 735/1,002)^4^	48.4%^5^ (*n* = 479/987)^6^	1.53	1.36, 1.72	<0.001
Adolescent girls (aged 15–19^1^)	76.6% (*n* = 193/252)	49.1% (*n* = 122/249)	1.58	1.37, 1.83	<0.001
Adolescent boys (aged 15–19^1^)	62.2% (*n* = 155/250)	27.9% (*n* = 70/249)	2.37	1.77, 3.17	<0.001
Women (aged 20–24^1^)	84.4% (*n* = 209/248)	65.7% (*n* = 163/247)	1.33	1.05, 1.69	0.021
Men (aged 20–24^1^)	70.3% (*n* = 178/252)	51.0% (*n* = 124/242)	1.41	1.15, 1.73	0.002

^1^Age at time of consent to receive a Yathu Yathu PPC.

^2^PR = prevalence ratio. Overall: adjusted for age, sex, and community. Each age-sex group: adjusted for community.

^3^Arithmetic mean of the 10 cluster-specific values of the proportion of AYP who knew their HIV status in the intervention arm.

^4^*n* = Number of individuals who knew their HIV status in the intervention arm, denominator = number of survey participants in the intervention arm.

^5^Arithmetic mean of the 10 cluster-specific values of the proportion of AYP who knew their HIV status in the control arm.

^6^*n* = Number of individuals who knew their HIV status in the control arm, denominator = number of survey participants in the control arm.

AYP, adolescents and young people; PPC, prevention points card.

### Impact on PrEP use, uptake of VMMC, and current use of ART

Among AYP self-reporting testing negative at their last HIV test, a higher percentage in the intervention arm had heard of PrEP compared to the control arm (37.9% versus 29.4%; *p* = 0.05). However, use was low in both trial arms. Overall, only 5% (*n* = 29) of AYP reported ever being offered PrEP and 0.4% (*n* = 7) reported ever taking PrEP in the last 12 months (Table 3).

**Table 3:** Impact of Yathu Yathu on uptake of HIV prevention and treatment services, 2021.

	Yathu Yathu arm	Control arm	Adjusted PR	95% CI	*p*-value
**Uptake of VMMC among adolescent boys and young men since September 2019 (*N* = 593)** ^1^	9.8%^5^ (*n* = 27/289)^6^	11.2%^7^ (*n* = 33/304)^8^	0.90	0.46, 1.78	0.76
Adolescent boys (aged 15–19^2^)	12.1% (*n* = 17/147)	14.1% (*n* = 21/158)	0.84	0.44, 1.62	0.59
Men (aged 20–24^2^)	7.1% (*n* = 10/142)	8.4% (*n* = 12/146)	0.94	0.51, 1.76	0.84
**HIV–negative AYP who report PrEP use in the last 12 months (*N* = 1,747)** ^3^	0.4% (*n* = 4/925)	0.4% (*n* = 3/822)	-	-	-
**HIV–positive AYP who report current use of ART (*N* = 48)** ^4^	92.9% (*n* = 26/28)	95.0% (*n* = 19/20)	-	-	-

^1^*N* = Number of adolescent boys and young men who were not circumcised at the start of the trial.

^2^Age at time of consent to receive a Yathu Yathu PPC.

^3^*N* = Number who self-reported their HIV status as HIV–negative in the endline survey.

^4^*N* = Number who self-reported they were HIV–positive in the endline survey. Overall: adjusted for age group and community. Each age group: adjusted for community.

^5^Arithmetic mean of the 10 cluster-specific values of the proportion of AYP with the outcome in the intervention arm.

^6^*n* = Number of individuals with the outcome in the intervention arm, denominator = number of survey participants included in the analysis in the intervention arm.

^7^Arithmetic mean of the 10 cluster-specific values of the proportion of AYP with the outcome in the control arm.

^8^*n* = Number of individuals with the outcome in the control arm, denominator = number of survey participants included in the analysis in the control arm.

ART, antiretroviral therapy; AYP, adolescents and young people aged 15–24; PPC, prevention points card; PR, prevalence ratio; PrEP, pre-exposure prophylaxis; VMMC, voluntary medical male circumcision.

Overall, 46.8% (*n* = 460/983) of ABYM reported ever being circumcised. Among ABYM not circumcised before the implementation of Yathu Yathu (*N* = 593), there was no evidence for a difference between trial arms in uptake of VMMC services, with 9.8% (*n* = 27/289) of ABYM in the intervention and 11.2% (*n* = 33/304) in the control arm reporting being circumcised since the implementation of Yathu Yathu (adjPR = 0.90 95% CI 0.46, 1.78; *p* = 0.76).

Among the 1,802 AYP who reported ever-testing for HIV, 2.7% self-reported living with HIV (*n* = 28 (2.8%) in the intervention arm and *n* = 20 (2.0%) in the control arm). By sex, 3.8% (*n* = 35/922) of AGYW and 1.5% (*n* = 13/880) of ABYM reported living with HIV. Among these AYP, almost all (94%; *n* = 45/48) were currently taking ART, with no evidence of a difference in current use between trial arms (Table 3).

### Impact on condom use, met need for contraceptive services, and pregnancy

Among all AYP, 75.7% (*n* = 1,505) reported ever having sex, among whom 73.4% (*n* = 1,105) reported sex in the last 12 months. The percentage of AYP who reported ever having sex in the last 12 months was similar in the intervention and control arms (74.8% versus 71.7%, respectively). Among these AYP, there was no evidence that Yathu Yathu had an impact on condom use at last sex (40.4%, *n* = 231/566 versus 41.5%, *n* = 221/538, respectively, adjPR = 0.97 95% CI 0.80, 1.18; Table 4). Results were similar by sex (Table 4); however, condom use differed by sex and age, with use lowest among women aged 20 to 24.

**Table 4:** Impact of Yathu Yathu on access to contraceptives and pregnancy prevention among AGYW, 2021.

	Yathu Yathu arm	Control arm	Adjusted PR^2^	95% CI	*p*-value
**Condom use at last sex among AYP reporting ever having had sex in the last 12 months (*N* = 1,104)** ^3^	40.4%^6^ (*n* = 231/566)^7^	41.5%^8^ (*n* = 221/538)^9^	0.97	0.80, 1.18	0.76
Adolescent girls (aged 15–19)^1^	40.6% (*n* = 51/118)	49.8% (*n* = 58/115)	0.76	0.52, 1.10	0.14
Adolescent boys (aged 15–19)^1^	59.2% (*n* = 50/90)	63.3% (*n* = 47/72)	0.86	0.59, 1.26	0.41
Women (aged 20–24)^1^	26.8% (*n* = 51/192)	26.0% (*n* = 49/193)	1.00	0.76, 1.31	0.99
Men (aged 20–24)^1^	47.6% (*n* = 79/166)	43.6% (*n* = 67/158)	1.08	0.87, 1.34	0.47
**Met need for contraceptives among sexually active AGYW (*N* = 507)** ^4^	60.1% (*n* = 148/249)	59.7% (*n* = 155/258)	1.02	0.79, 1.31	0.89
Adolescent girls (aged 15–19)^1^	55.4% (*n* = 48/94)	47.8% (*n* = 48/100)	1.30	0.69, 2.42	0.39
Young women (aged 20–24)^1^	64.1% (*n* = 100/155)	66.9% (*n* = 107/158)	0.95	0.76, 1.19	0.67
**Proportion of all AGYW reporting a birth in the last 12 months (*N* = 987)** ^5^	12.6% (*n* = 62/494)	12.4% (*n* = 61/493)	1.02	0.60, 1.72	0.95
Adolescent girls (aged 15–19)^1^	9.6% (*n* = 24/248)	7.4% (*n* = 18/246)	1.20	0.58, 2.46	0.60
Young women (aged 20–24)^1^	15.5% (*n* = 38/246)	17.4% (*n* = 43/247)	1.06	0.58, 1.91	0.85
**Proportion of AGYW reporting a birth in the last 12 months among AGYW who report having had sex in the last 12 months (*N* = 780)** ^6^	16.1% (*n* = 62/390)	15.4% (*n* = 61/390)	1.01	0.61, 1.69	0.95
Adolescent girls (aged 15–19)^1^	15.7% (*n* = 24/159)	11.2% (*n* = 18/161)	1.24	0.58, 2.65	0.56
Young women (aged 20–24)^1^	16.5% (*n* = 38/231)	18.3% (*n* = 43/229)	1.04	0.60, 1.82	0.88

^1^Age at time of consent to receive a Yathu Yathu PPC.

^2^PR = Prevalence ratio. Overall: adjusted for age, sex, and community. Each age-sex group: adjusted for community.

^3^*N* = Number who self-reported any sex in the last 12 months.

^4^*N* = Number of AGYW who self-reported ever having had sex.

^5^*N* = all AGYW.

^6^*N* = Number of AGYW who self-reported any sex in the last 12 months. Arithmetic mean of the 10 cluster-specific values of the proportion of AYP with the outcome in the intervention arm.

^7^*n* = Number of individuals with the outcome in the intervention arm, denominator = number of survey participants included in the analysis in the intervention arm.

^8^Arithmetic mean of the 10 cluster-specific values of the proportion of AYP with the outcome in the control arm.

^9^ *n* = Number of individuals with the outcome in the control arm, denominator = number of survey participants included in the analysis in the control arm.

AGYW, adolescent girls and young women; AYP, adolescents and young people aged 15–24; PPC, prevention points card; PR, prevalence ratio.

Over three-quarters (79.0%; *n* = 787/996) of AGYW reported ever having sex, among whom 78.5% (*n* = 618) reported sex in the last 12 months. Almost 20% (18.0%) of these AGYW also reported wanting to have a/another child within the year. Among the AGYW who reported sex in the last 12 months and wanted to wait to have a/another child or did not want another child (or were unsure about when they wanted a child), around 60% reported current use of contraceptives, with no difference in met need between trial arms (60.1%, *n* = 148/249 versus 59.7%, *n* = 155/258, respectively; adjPR = 1.02 95% CI 0.79, 1.31; *p* = 0.89; Table 4).

Among AGYW who reported ever having had sex, there was no evidence that Yathu Yathu had an impact on births. In both trial arms, approximately 12% of AGYW reported a birth in the last 12 months (12.6%, *n* = 62/494 versus 12.4%, *n* = 61/493, respectively; adjPR = 1.02; 95% CI 0.60, 1.72, *p* = 0.95). The prevalence ratios comparing trial arms for met need for contraceptives and births in the previous 12 months were similar when stratified by age group, though the proportions with each outcome were higher among young women than adolescent girls (Table 4).

### Sensitivity analyses

Findings from sensitivity analyses were similar to those in the primary analyses (S1 and S2 Tables).

## Discussion

Delivery of community-based, peer-led, and incentivised SRH services to AYP was effective in increasing knowledge of HIV status compared to the control arm, with the largest effect among adolescent boys aged 15 to 19. Despite a large impact on this primary outcome, there was no evidence of impact on linkage to HIV prevention or care services nor evidence for an impact on met need for contraceptive services, or births, among AGYW. Our findings demonstrate that Yathu Yathu provides an important opportunity to reach AYP with HIV testing services. Additional efforts are required to translate increased knowledge of HIV status into improved linkage to HIV prevention and care services and to increase AGYW’s met need for contraceptive services.

Delivery of HIV testing services through the Yathu Yathu strategy had a large impact on uptake of testing. This is consistent with results from the P-ART-Y study, which included the 2 Yathu Yathu study communities and showed substantially increased knowledge of HIV status among adolescents who had been offered door-to-door HIV testing services [25]. Our finding is also consistent with an impact evaluation study of the DREAMS Partnership in Kenya, where AGYW who were invited to participate in DREAMS were offered HIV testing at “safe spaces” in the community [19], and with an observational case study in South Africa [38] that assessed delivery of SRH services to AYP through a youth centre incentivised through rewards (that included socks, pens, and computer time). Consistent with these 2 studies, our findings also indicated a larger impact among adolescents than young adults, particularly adolescent boys. Reaching adolescent boys with HIV testing services provides an important opportunity to inform boys about SRH, refer them to VMMC services; with men less likely to test for HIV than women [39,40], early engagement with HIV testing services may help to overcome some barriers to HIV testing observed among men, including fear [41] potentially encouraging more frequent HIV testing in adulthood. Adolescents are more likely to be economically dependent than young adults; the reward system likely appeals to them because it provides an opportunity to access products that would otherwise be out of their reach. Provision of these products likely encourages adolescents, who may be less likely to consider SRH services important to them, to place greater value on these services.

Testing for HIV is the critical first step into the HIV care continuum or to HIV prevention services. Despite removing barriers to HIV prevention services, including a lack of youth-friendly services at health facilities, we found no impact on uptake of these services. Among the small number of AYP self-reporting their HIV–positive status, coverage of ART was high in both trial arms, suggesting that current ART services are performing well for young people who know their HIV–positive status. Our findings are similar to, yet higher than, those reported in the 2016 Population-based Impact Assessment (80%) [42]. These findings could reflect improved access to ART among AYP over time but also reflect the impact of the PopART and P-ART-Y study on access to ART and community-level perceptions regarding the importance of ART access for AYP [43]. A Yathu Yathu model could still complement existing treatment services, providing support for adherence and for transitioning to adult-centred care [44].

Few AYP reported ever using PrEP. Low use may be attributable, in part, to weak referral mechanisms between Yathu Yathu and the health facility, poor delivery of PrEP at the health facilities or low acceptability of PrEP among potential users. With barriers to existing health services well established among AYP [45,46] low use may also be attributable to barriers to accessing the health facility in general. Yathu Yathu hub staff did not make a direct offer of PrEP to AYP, but counselled AYP based on the result of their HIV test and referred them to the health facility for PrEP. A direct offer of on-site initiation of PrEP to AYP testing HIV–negative might have resulted in increased use. In Zimbabwe, a study with young women who sell sex found that PrEP use was associated with an active offer of PrEP [47]. In Kenya, programmatic data from 89 sites that offered PrEP found that few AGYW were screened for PrEP eligibility and this meant that they were not offered PrEP [48]. With evidence of the efficacy of injectable PrEP [49] and recommended use of the dapivirine ring followed by its approval for use in South Africa, future research should explore whether informing AYP about different formulations of PrEP [50]. Further, PrEP should be promoted through a narrative of the potential gains of PrEP use [51], such as enabling control over health [52]. Alongside a direct offer to initiate PrEP on-site and accurate information about PrEP, this could translate into higher uptake through a Yathu Yathu model.

The majority of ABYM who reported ever being circumcised had been circumcised before the implementation of Yathu Yathu, likely reflecting the national VMMC campaign launched by the Ministry of Health in 2007 and scaled up in 2015 [53]. By 2020, the target was to reach approximately 2 million boys and men aged 10 to 49 with VMMC services [54]. Three million circumcisions were performed, the majority among boys aged 10 to 19 [55]. Studies have shown that increasing VMMC coverage among young men, as compared with boys, in particular is challenging [56]. To increase coverage, more intensive promotion of the benefits of VMMC and onsite services are likely required to reach ABYM less certain about the potential HIV prevention and other health-related benefits of circumcision [57]. In our study, one-third of ABYM who ever had sex reported their first sexual experience by age 15 years. Future implementation of the Yathu Yathu strategy should consider providing selected SRH services to boys aged under 15 years and provide VMMC on site.

We found no evidence that Yathu Yathu increased condom use at last sex. Condom use is affected by several factors [58]; a systematic review of 23 studies shows that suggesting condom use with a regular partner can raise issues of trust and condoms are seen as reducing sexual pleasure, particularly for men [58]. For AGYW, negotiating condom use can be challenging, particularly where there are power imbalances in their relationships [59]. The provision of information on condoms and condom use and distribution of condoms is likely insufficient to change these broader, structural factors that affect condom use. Low condom use among women aged 20 to 24 years may also reflect fertility desires and expectations. Similarly, we found no evidence for an impact on met need for contraceptives or birth rates. With hubs closed for 3 months in response to COVID-19 in mid-2020 and stock-outs of oral contraceptives at the hubs and health facilities between 2020 and 2021, it is possible that some AGYW discontinued use of hormonal contraceptives, particularly the pill, and subsequently lost confidence that services would be offered consistently. Absence of an effect may also reflect norms surrounding pregnancy and fertility, which are complex and which Yathu Yathu was unlikely to change during the relatively short implementation period [60]. A component of Yathu Yathu was community mobilisation to address norms related to AYP’s access to condoms and contraceptives that were anticipated to hinder service use. Early experience suggested that these mobilisation activities were insufficient to shift these norms. In response, a community dialogue approach was later implemented to discuss community perspectives on the need for contraceptive services for AYP in the context of high levels of unintended pregnancies [61].

Our trial had limitations. Approximately 35% of AYP randomly selected for participation in the endline survey could not be contacted, due either to moving (out of the zone in which they were resident in 2019, or out of the community) or to not being contactable at the time of the survey. If these individuals were more or less likely to know their HIV status compared with survey participants, for example, if mobile individuals were less likely to have accessed the Yathu Yathu hubs than stable residents, our findings may be biased. However, as the difference between trial arms in our primary outcome was large, it is probable that we would still have identified an intervention effect even if these individuals (who could not be contacted) had been included in the survey. Furthermore, the participation rate in the survey among AYP who remained resident in their zones and therefore eligible for participation in the survey was high, and similar in both study arms.

Although we were concerned our trial might be subject to contamination, with AYP in control zones able to access services at the hubs in intervention zones, we found little evidence that AYP in control zones attended the hubs. All our outcomes were based on self-report and are subject to social desirability bias. Responses in the Yathu Yathu arm may have been more subject to this bias, as AYP learnt of the importance of HIV testing through Yathu Yathu. The teams conducting the survey were different from those delivering the services and the AYP were unaware of the primary outcome of the trial, both should have limited this bias. Delivery of Yathu Yathu was affected by COVID-19, with implementation halted over 3 months, and after July 2020, implementation was adapted to limit the number of individuals allowed to attend the hubs at any given time. These restrictions, uncertainty among AYP as to whether the hubs would be closed permanently and fear of COVID-19 infection at the hubs may have reduced AYP’s willingness and ability to access Yathu Yathu services and may have implications for our findings. In the absence of the pandemic, we might have observed higher uptake of services and a greater effect of Yathu Yathu on secondary outcomes.

Despite limitations, Yathu Yathu had a large impact on knowledge of HIV status and reached a large proportion of AYP participating in the endline survey. With adolescents in particular experiencing barriers to accessing health facilities and given the large observed effect on knowledge of HIV status among young men, delivery of HTS through community-based hubs should be made available to adolescents and considered in settings where health facilities are failing to reach young men. The lack of an effect on the secondary outcomes, many of which reflect services available through linkage to the health facility, likely reflects known barriers to accessing the health facility; findings that are similar to a study in Malawi, which found higher uptake of SRH services when delivered through youth-friendly services [62]. Implementation research is needed to assess whether delivery of PrEP and VMMC on-site increases service use, the extent to which context affects AYP’s access to and use of the hubs, particularly contraceptive services, and how to shift norms in support of AYP’s continued and increased use of SRH services. In addition, research on the long-term sustainability of the model and the feasibility of integrating services for other health outcomes, including support for mental health, should be considered.

Our CRT provides rigorous evidence that delivery of community-based, peer-led, and incentivised SRH services by peers is feasible, even in the context of a pandemic. We showed that the strategy reaches a high proportion of AYP and is highly effective in increasing knowledge of HIV status among a population underserved by available health services. Even in the context of COVID-19, essential SRH services continued to be delivered through Yathu Yathu with adaptations to implementation [32]. We consider the findings generalisable to areas with both high HIV prevalence and where AYP are known to have less access to SRH services and to areas previously exposed to community-based delivery of HIV testing services. A critical next step is to estimate the cost-effectiveness of the strategy and to implement and evaluate streamlined iterations of Yathu Yathu, with greater emphasis on linkage to services after HIV testing, in order to have a greater impact on the health and well-being of AYP.

## Supporting information

S1 CONSERVE Checklist CONSERVE-CONSORT 2021 checklist. (DOCX)

S1 CONSORT Checklist CONSORT 2010 checklist. (DOCX)

S1 Table Knowledge of HIV status among adolescents and young people, by arm, 2021. (DOCX)

S2 Table Impact of Yathu Yathu on access to contraceptives and pregnancy prevention among AGYW, 2021. (DOCX)

S1 Inclusivity in Global Research Questionnaire Inclusivity in global research 2021 questionnaire. (DOCX)

## Abbreviations

ABYM: adolescent boys and young men
adjPR: adjusted prevalence ratio
AGYW: adolescent girls and young women
ART: antiretroviral therapy
AYP: adolescents and young people
COVID-19: Coronavirus Disease 2019
CRT: cluster-randomised trial
CSE: comprehensive sexuality education
PPC: prevention points card
PR: prevalence ratio
PrEP: pre-exposure prophylaxis
SRH: sexual and reproductive health
VMMC: voluntary medical male circumcision
