# Supplementary material for: The impact of community-based, peer-led sexual and reproductive health services on knowledge of HIV status among adolescents and young people aged 15 to 24 in Lusaka, Zambia: The Yathu Yathu cluster-randomised trial
Source: PLoS Med. 2023 Apr 21;20(4):e1004203. doi: 10.1371/journal.pmed.1004203 (PMC10121029; doi:10.1371/journal.pmed.1004203)
Supplement: S1 Table — (DOCX) [file pmed.1004203.s003.docx]

**S1 Table. Knowledge of HIV status among adolescents and young people, by arm, 2021**

|  | Yathu Yathu Arm | Control Arm | Adjusted PR^2^ | 95% CI | p-value |
| --- | --- | --- | --- | --- | --- |
| Sensitivity analysis 1 – adjustment for community, age, sex, education and marital status | | | | | |
| Overall | 73.3%^3^  (n=735/1002)^4^ | 48.4%^5^  (n=479/987)^6^ | 1.53 | 1.36, 1.72 | <0.001 |
| Adolescent girls (aged 15-19^1^) | 76.6%  (n=193/252) | 49.1%  (n=122/249) | 1.57 | 1.36,  1.80 | <0.001 |
| Adolescent boys (aged 15-19^1^) | 62.2%  (n=155/250) | 27.9%  (n=70/249) | 2.33 | 1.74,  3.12 | <0.001 |
| Women (aged 20-24^1^) | 84.4%  (n=209/248) | 65.7%  (n=163/247) | 1.33 | 1.05,  1.69 | 0.020 |
| Men (aged 20-24^1^) | 70.3%  (n=178/252) | 51.0%  (n=124/242) | 1.39 | 1.12,  1.73 | 0.005 |
| Sensitivity analysis 2 – Control arm individuals who visited the hubs and knew their HIV status, assume they would not have known their HIV status in absence of the hub | | | | | |
| Overall | 73.3%^3^  (n=735/1002)^4^ | 46.0%  (n=455/987) | 1.61 | 1.43, 1.80 | <0.001 |
| Adolescent girls (aged 15-19^1^) | 76.6%  (n=193/252) | 45.5%  (n=113/249) | 1.72 | 1.46,  2.03 | <0.001 |
| Adolescent boys (aged 15-19^1^) | 62.2%  (n=155/250) | 25.5%  (n=64/249) | 2.64 | 1.92,  3.65 | <0.001 |
| Women (aged 20-24^1^) | 84.4%  (n=209/248) | 64.1%  (n=159/247) | 1.37 | 1.08,  1.73 | 0.013 |
| Men (aged 20-24^1^) | 70.3%  (n=178/252) | 48.9%  (n=119/242) | 1.46 | 1.22,  1.74 | <0.001 |

^1^Age at time of consent to receive a Yathu Yathu prevention points card (PPC), ^2^PR = Prevalence ratio. Overall: adjusted for age, sex and community. Each age-sex group: adjusted for community; ^3^ Arithmetic mean of the 10 cluster-specific values of the proportion of AYP who knew their HIV status in the intervention arm; ^4^ n=number of individuals who knew their HIV status in the intervention arm, denominator=number of survey participants in the intervention arm; ^5^ Arithmetic mean of the 10 cluster-specific values of the proportion of AYP who knew their HIV status in the control arm; ^6^ n=number of individuals who knew their HIV status in the control arm, denominator=number of survey participants in the control arm.
