# Supplementary material for: The impact of community-based, peer-led sexual and reproductive health services on knowledge of HIV status among adolescents and young people aged 15 to 24 in Lusaka, Zambia: The Yathu Yathu cluster-randomised trial
Source: PLoS Med. 2023 Apr 21;20(4):e1004203. doi: 10.1371/journal.pmed.1004203 (PMC10121029; doi:10.1371/journal.pmed.1004203)
Supplement: S2 Table — (DOCX) [file pmed.1004203.s004.docx]

**S2 Table. Impact of Yathu Yathu on contraceptives and pregnancy prevention among AGYW, 2021**

|  | Yathu Yathu Arm | Control Arm | Adjusted PR^2^ | 95%CI | p-value |
| --- | --- | --- | --- | --- | --- |
| Condom use at last sex among AYP reporting ever having had sex in the last 12-months (N=1054) | 40.5%  (n=211/516) | 41.5%  (n=221/538) | 0.96 | 0.77, 1.20 | 0.70 |
| Adolescent girls (aged 15-19^1^) | 40.3%  (n=49/114) | 49.8%  (n=58/115) | 0.75 | 0.52, 1.09 | 0.12 |
| Adolescent boys (aged 15-19^1^) | 61.7%  (n=48/82) | 63.3%  (n=47/72) | 0.89 | 0.61, 1.30 | 0.54 |
| Women (aged 20-24^1^) | 25.2%  (n=42/171) | 26.0%  (n=49/193) | 0.92 | 0.66, 1.28 | 0.58 |
| Men (aged 20-24^1^) | 48.4%  (n=72/149) | 43.6%  (n=67/158) | 1.08 | 0.83, 1.40 | 0.54 |
| Met need for contraceptives among sexually active AGYW (N=489) | 59.8%  (n=136/231) | 59.7%  (n=155/258) | 1.02 | 0.79,  1.31 | 0.87 |
| Adolescent girls (aged 15-19^1^) | 55.0%  (n=47/92) | 47.8%  (n=48/100) | 1.29 | 0.70,  2.39 | 0.39 |
| Young women (aged 20-24^1^) | 64.3%  (n=89/139) | 66.9%  (n=107/158) | 0.96 | 0.77,  1.20 | 0.70 |
| Proportion of all AGYW reporting a pregnancy in the last 12 months (N=950) | 12.6%  (n=57/457) | 12.4%  (n=61/493) | 1.03 | 0.61,  1.75 | 0.91 |
| Adolescent girls (aged 15-19^1^) | 9.5%  (n=23/239) | 7.4%  (n=18/246) | 1.17 | 0.56,  2.43 | 0.66 |
| Young women (aged 20-24^1^) | 15.9%  (n=34/218) | 17.4%  (n=43/247) | 1.07 | 0.59,  1.95 | 0.80 |
| Proportion of AGYW reporting a pregnancy in the last 12 months among AGYW who report having had sex in the last 12 months (N=746) | 16.4%  (n=57/356) | 15.4%  (n=61/390) | 1.03 | 0.61,  1.74 | 0.90 |
| Adolescent girls (aged 15-19^1^) | 15.5%  (n=23/153) | 11.2%  (n=18/161) | 1.21 | 0.56,  2.61 | 0.61 |
| Young women (aged 20-24^1^) | 17.0%  (n=34/203) | 18.3%  (n=43/229) | 1.07 | 0.61,  1.87 | 0.81 |

^1^ Age at time of consent to receive a Yathu Yathu prevention points card (PPC); PR = Prevalence ratio. Overall: adjusted for age, sex and community. Each age-sex group: adjusted for community;
